# Contrasting predictors of poor antiretroviral therapy outcomes in two South African HIV programmes: a cohort study

**DOI:** 10.1186/1471-2458-10-430

**Published:** 2010-07-22

**Authors:** Mison Dahab, Salome Charalambous, Alan S Karstaedt, Katherine L Fielding, Robin Hamilton, Lettie La Grange, Gavin J Churchyard, Alison D Grant

**Affiliations:** 1 Clinical Research Unit, Department of Infectious and Tropical Diseases, London School of Hygiene & Tropical Medicine, London, UK; 2 Aurum Institute, Johannesburg, South Africa; 3 Collaborative Programme for AIDS Research in South Africa, University of KwaZulu-Natal, Durban, South Africa; 4 Adult HIV Clinic, Chris Hani Baragwanath Hospital, Johannesburg, South Africa; 5 Infectious Disease Epidemiology Unit, Department of Epidemiology and Public Health, London School of Hygiene & Tropical Medicine, London, UK; 6 Anglo Platinum, Safety, Health & Environment, Johannesburg, South Africa

## Abstract

**Background:**

Many national antiretroviral therapy (ART) programmes encourage providers to identify and address baseline factors associated with poor treatment outcomes, including modifiable adherence-related behaviours, before initiating ART. However, evidence on such predictors is scarce, and providers judgement may often be inaccurate. To help address this evidence gap, this observational cohort study examined baseline factors potentially predictive of poor treatment outcomes in two ART programmes in South Africa, with a particular focus on determinants of adherence.

**Methods:**

Treatment-naïve patients starting ART were enrolled from a community and a workplace ART programme. Potential baseline predictors associated with poor treatment outcomes (defined as viral load > 400 copies/ml or having discontinued treatment by six months) were assessed using logistic regression. Exposure variables were organised for regression analysis using a hierarchical framework.

**Results:**

38/227 (17%) of participants in the community had poor treatment outcomes compared to 47/117 (40%) in the workplace. In the community, predictors of worse outcomes included: drinking more than 20 units of alcohol per week, having no prior experience of chronic medications, and consulting a traditional healer in the past year (adjusted odds ratio [aOR] 15.36, 95% CI 3.22-73.27; aOR 2.30, 95%CI 1.00-5.30; aOR 2.27, 95% CI 1.00-5.19 respectively). Being male and knowing someone on ART were associated with better outcomes (aOR 0.25, 95%CI 0.09-0.74; aOR 0.44, 95%CI 0.19-1.01 respectively). In the workplace, predictors of poor treatment outcomes included being uncertain about the health effects of ART and a traditional healer's ability to treat HIV (aOR 7.53, 95%CI 2.02-27.98; aOR 4.40, 95%CI 1.41-13.75 respectively). Longer pre-ART waiting time (2-12 weeks compared to <2 weeks) predicted better treatment outcomes (aOR 0.13, 95% CI 0.03-0.56).

**Conclusion:**

Baseline predictors of poor treatment outcomes were largely unique to each programme, likely reflecting different populations and pathways to HIV care. In the workplace, active promotion of HIV testing may have extended ART to individuals who, without provider initiation, would not have spontaneously sought care. As provider-initiated testing makes ART available to individuals less motivated to seek care, patients may need additional adherence support, especially addressing uncertainty about the health benefits of ART.

## Background

The global burden of HIV is heaviest in lower-income countries where the majority of adults with HIV live [1]. However by 2008 only 42% of those in need of antiretroviral therapy (ART) worldwide were receiving treatment [2]. The goal of ART is for patients to achieve and maintain virological suppression for as long as possible. In many low-income countries, where access to ART was very limited prior to the availability of highly efficacious triple combination therapy, the most important determinant of virological suppression is adherence to clinical follow-up and to treatment [3-8]. Contrary to initial scepticism, good treatment outcomes including both virological response and self-reported adherence were reported in early pilot ART programmes in lower-income countries, compared to high-income country programmes [6,9-11].

Despite these encouraging early results, declining levels of adherence and poorer treatment outcomes have been reported in some lower-income countries as ART programmes have been scaled up [12-16]. Little is known about the reasons for worsening outcomes in these programmes, and since the majority of published literature represents studies that are cross-sectional in design, even less is known about baseline factors predictive of poor treatment outcomes. Many ART guidelines emphasise the need for providers to identify and address risk factors for poor adherence, and providers routinely use their own judgement, thought to be no better than chance [17,18], to predict future adherence behaviours. Given the importance of appropriately supporting patients starting ART, it is vital to identify baseline behavioural factors related to adherence that influence poor treatment outcomes. In this cohort study we examined baseline factors potentially predictive of poor treatment outcomes, defined as unsuppressed viral load and complete discontinuation of clinical follow-up and treatment, in two established programmes in South Africa, one a community public-sector programme and one a workplace-based programme. The focus was on factors potentially influencing adherence to treatment and to clinical follow-up, with a view to identifying individuals who might benefit from more intensive adherence support.

## Methods

### Setting

This study was conducted within two ART programmes in South Africa. The first was a community programme located within a tertiary public-sector hospital serving a diverse peri-urban population in Johannesburg. Most patients self-presented for HIV treatment after undergoing voluntary counselling and testing (VCT) in a primary health centre. HIV treatment and care, including laboratory testing and treatment for opportunistic infections, were provided free of charge but patients were expected to cover other expenses such as transport to the clinic. Individuals were medically eligible for ART if they were in WHO stage 4 and/or had a CD4 count of <200 cells/mm^3^. The first line ART regimen was d4T/3TC/efavirenz (or nevirapine if efavirenz was contraindicated).

The second study site was a workplace programme located within a tertiary mining company hospital in Northwest Province. Most patients were referred for treatment after undergoing counselling and testing as part of periodic VCT campaigns, yearly employment health examinations or provider-initiated testing and counselling (PITC). The hospital provided free HIV treatment and care, including laboratory testing and treatment for opportunistic infections, as well as free transport to the clinic for mine employees. Individuals were medically eligible for ART if they were in WHO stage 4; or had a CD4 count of <250 cells/mm^3^; or were in WHO stage 3 with a CD4 count <350 cells/mm^3^. The first line ART regimen was Combivir (AZT + 3TC) and efavirenz (or nevirapine if efavirenz was contraindicated).

### Design and participants

This cohort study was conducted among consenting individuals initiating ART in the two study sites. Participants had to be at least 18 years of age, ART naïve, medically eligible for ART initiation, and willing to give written informed consent for enrolment. In both sites, clinic staff were asked to refer individuals presenting to start ART to the research study staff. The clinic staff did not collect information on the number of individuals starting ART who were eligible for the study, nor those among them who refused referral to the research staff.

### Study procedures

At baseline, a semi-structured questionnaire taking approximately 45 minutes was administered to participants at both sites to assess exposures potentially predictive of poor adherence. Participants were also asked to provide contact information in case they missed the study follow-up visit. Refusal to provide this information was not an exclusion criterion. At the six-week programme visit, participants were interviewed briefly for an interim assessment of self-reported adherence. At the six-month programme visit, conducted between 20 and 28 weeks after starting ART, a short questionnaire concerning modifiable behavioural factors potentially related to adherence was administered, and viral load measurements were obtained from the clinical records. Those who missed their six-month visit by one month or more were classified as having discontinued care. They were contacted using previously collected locator information, and a semi-structured questionnaire was conducted to ascertain treatment outcomes. This was done in person whenever possible, but telephonically otherwise, to confirm reasons for discontinuation of care. All study interviews were conducted by a trained research nurse in the participant's preferred language (whether English or one of the four main local languages).

### Exposures of interest

**Figure 1:**
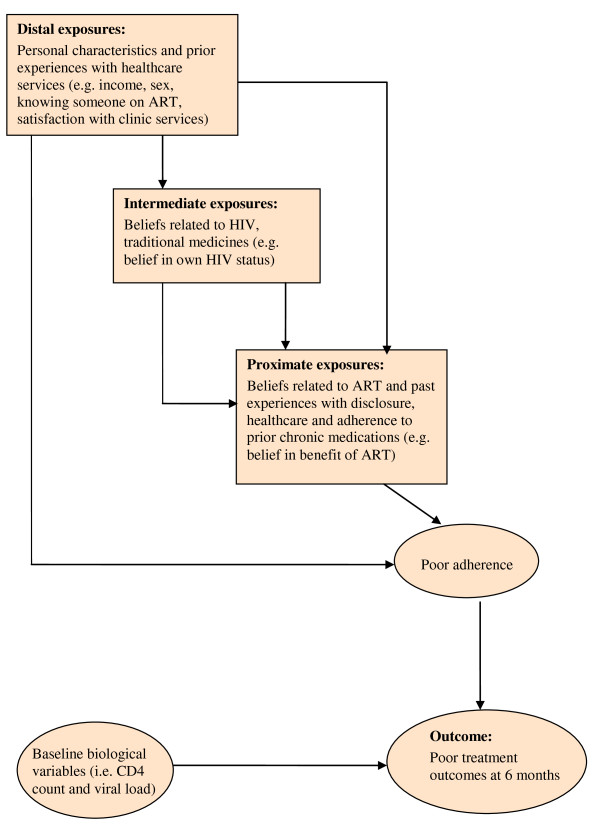
**Hierarchical conceptual framework for logistic regression**. An illustration of the hierarchical conceptual framework used to conduct the risk factor analysis where factors of interest were organised into three levels according to how directly they were thought to influence adherence behaviour.

The choice of exposures of interest assessed at baseline was informed by preliminary pilot work conducted in the workplace programme [19]. Exposure variables were organised according to a hierarchical conceptual framework (see Figure 1), within which factors of interest were grouped into three levels according to how directly they were hypothesised to influence adherence behaviour.

### Outcome

In the primary analysis participants were classified as having a poor treatment outcome if: (i) they attended the six-month programme visit but had an HIV viral load >400 copies/ml; or (ii) they missed the six-month visit by one month or more, and upon tracing were found to be alive but no longer taking ART.

Since our main interest was in factors affecting adherence, individuals known to have died before six months were excluded from the analysis. This is because early mortality on ART is more likely due to advanced disease, and co-morbidity at baseline, than to poor adherence [20,21]. Also excluded were participants who missed their six-month visit, but could not be traced to confirm their treatment outcomes.

### Sample size

Based on recruitment rates in the study programmes prior to the start of the study, 400 patients was the maximum number of participants feasibly expected to be recruited during the allotted study recruitment period. The overall percentage of patients failing to achieve virological suppression at six months among ART patients in the workplace programme was estimated at 37% (Charalambous S, unpublished data). For an overall power of 90% and a type 1 error of 5%, the sample size of 400 would have enabled the detection of an odds ratio of 4 for any risk factor with a prevalence of 10-15% and an odds ratio of 3 for any risk factor with a prevalence of 25-35% in the population. A sample size of 300 would have enabled the detection of an odds ratio of 4 for any risk factor with a prevalence of 15-35%.

### Data analysis

Data analysis was performed with Stata 10 software (Stata Corp., College Station, Texas, USA). The unadjusted association of each factor with the outcome was expressed as an odds ratio (OR) and its associated 95% confidence interval (CI) and p-value from the chi-square or Fisher's exact test. Variables that showed evidence of an association with the outcome in the univariable analysis (p < 0.2), and any *a priori *confounders, were examined in the multivariable analysis.

Logistic regression analysis was conducted to assess associations between exposures of interest and the outcome. Exposures of interest were analysed using a hierarchical framework that grouped exposures into three main levels: distal, intermediate and proximate, depending on how directly they were thought to influence the outcome (see Figure 1 and Appendix 1). The framework served to better organise the analysis in order to avoid the misclassification of proximate factors as confounders of the distal ones [22] and to enable the grouping of similar variables into the same hierarchical level in order to assess co-linearity. In the case of ordered categorical variables, departures from linearity were assessed and, when appropriate, a test for linear trend was conducted.

The multivariable model was constructed in three stages. An initial model was constructed using distal variables that showed evidence of an association with the outcome in the univariable analysis (p < 0.2) and age, considered as an *a priori *confounder. Next, an intermediate-level model was constructed by adding the intermediate variables retained from the univariable analysis. Lastly, the final model was constructed by adding the proximate variables of interest. Explanatory variables were sequentially dropped within each group, starting with variables with the weakest association with the outcome, based on the likelihood ratio test. Only variables associated with the outcome with p < 0.1 were retained in each model.

A sensitivity analysis was conducted using the more commonly used proxy measure of poor adherence, having a viral load of >400 copies/ml, restricted to individuals with an available viral load result at the six-month visit, in order to determine whether the models would change when the outcome of discontinuing treatment was excluded. A second sensitivity analysis was conducted to test the effect of an alternative hierarchical order than that employed in the primary conceptual framework. Variables strongly associated with the outcome in the primary framework were moved from the distal to the proximate level or vice-versa.

### Ethics

The study was approved by the Research Ethics Committee of the University of KwaZulu-Natal, the Human Ethics Research Committee of the University of Witwatersrand, and the Ethics Committee of the London School of Hygiene & Tropical Medicine, UK. Each participant provided written informed consent for study participation.

## Results

### Participants

Enrolment of study participants took place between May 2006 and February 2007. Follow-up continued until September 2007. During the study period the total number of patients who started ART, irrespective of whether or not they met the study eligibility criteria, was 550 in the community programme and 216 in the workplace programme. Of this population 267 and 144 participants were enrolled into the study in the community and the workplace respectively. The gender and age profiles of the general patient population and of the study cohort were similar. In the community, 64% of the general patient population were female compared to 61% of study participants, while the median age of the general patient population was 38 years, compared to 37.5 years for participants. In the workplace, 5% of both general patient population and those enrolled were female, while the median age in both groups was 46 years.

**Figure 2:**
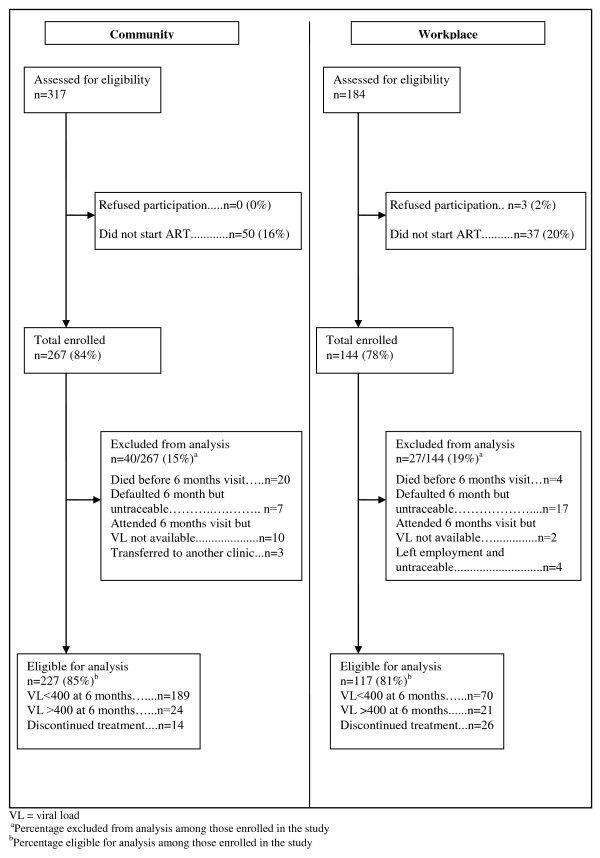
**Cohort construction by programme**. (i) Total number of individuals assessed for eligibility for enrolment in the cohort study; (ii) proportion of those assessed enrolled in the cohort study in each site; (iii) proportion of enrolled participants included in the risk factor analysis and their treatment outcomes; (iv) percentage excluded from the analysis and reasons for exclusion.

Figure 2 shows the construction of the study cohort. Among those enrolled in the community programme, 40/267 (15%) were excluded from the risk factor analysis mainly due to death and loss to follow up with unknown treatment outcomes. In the workplace 27/144 (19%) were excluded, primarily because they could not be traced to confirm treatment outcomes. All participants agreed to provide contact information for later tracing in case they missed the six-month visit.

Characteristics of the 227 community and 117 workplace participants included in the risk factor analysis are shown in Table 1. In the community programme the median age was 37 years (interquartile range [IQR] 31-44), and the majority were female (67%) and unemployed (71%), despite a median duration in education of 11 years (IQR 8-12). The median CD4 count at ART start was 101 cells/mm^3^. The 40 individuals in the community programme excluded from the risk factor analysis were similar in age (median 38 years) and educational level (median 11 years) to those included, but were less likely to be female (47%) and had a lower baseline CD4 count (median 66 cells/mm^3^). In the workplace, only six (5.1%) were female. The median age was 46 years (IQR 37-52), median duration in education was seven years (IQR 3-9), and median CD4 count was 183 cells/mm^3^. The 27 individuals excluded from the risk factor analysis were similar to those included in age (median 47 years), sex (none female), educational level (median 4 years) and baseline CD4 count (median 162 cells/mm^3^).

**Table 1:** Distribution of selected variables at start of antiretroviral therapy, by programme

**Variable**	**Community** **n = 227**	**Workplace** **n = 117**
		
Female, n (%)	152 (67.0%)	6 (5.1%)
		
Age, median (IQR), in years	37 (31-44)	46 (37-52)
		
Education, median (IQR), in years	11 (8-12)	7 (3-9)
		
Unemployed, n (%)	161(70.9%)	2 (1.7%)
		
Clinic waiting time, median (IQR), in hours	6 (4-7)	4 (4-5)
		
Time since HIV diagnosis, median (IQR), in months	11.7 (4.4-37.0)	1.4 (0.7-11.9)
		
CD4 count at baseline, median (IQR) in cells/mm^3^	101 (46-154)	183 (125-237)

IQR = interquartile range

### Outcome assessment

In the community programme six months after ART start, 38/227 (16.7%) had poor treatment outcomes (Figure 2). This included 24 (10.6%) who did not achieve virological suppression at six months and an additional 14 (6.2%) who discontinued treatment and care altogether. By contrast, in the workplace programme 47/117 (40.2%) had poor treatment outcomes. This included 21 (18.0%) who did not achieve virological suppression and 26 (22.2%) who discontinued treatment.

### Predictors of poor treatment outcomes

Table 2 shows the results of the univariable analysis organised using the hierarchical conceptual framework with exposures grouped into distal, intermediate and proximate levels. Factors found to be associated with increased odds of poor treatment outcomes (p < 0.2) in the univariable analysis were largely unique to each programme. At the distal level in the community programme, these factors were being female, not knowing someone on ART, reporting having been tested for HIV without being informed, and high alcohol consumption. In the workplace, these were fewer years of education, a CD4 count at baseline of 100-200 cells/mm^3^, not knowing anyone who died of HIV, and a delay of less than two weeks between HIV diagnosis and ART initiation. Dissatisfaction with programme services was associated with poor treatment outcomes in both programmes. At the intermediate level, factors associated with increased odds of poor treatment outcomes in both programmes were uncertainty regarding the existence of HIV and one's own status. However, the prevalence of these beliefs was extremely low in the community programme. At the proximate level, in the community programme, not having been prescribed chronic treatments in the past was associated with poor treatment outcomes. In the workplace, uncertainty regarding the positive effects of ART, non-disclosure of HIV status, belief that ART can cure HIV and that ART adherence will be difficult, and sharing of medicines with family and friends in the past were associated with poor treatment outcomes.

**Table 2:** Univariable level predictors of poor treatment outcomes (viral load >400 copies/ml and complete discontinuation of follow-up and treatment six months post treatment initiation) on antiretroviral therapy in community and workplace programmes

**Variables of interest**	**Community**				**Workplace**			
	**n(%) poor treatment outcomes**	**Unadjusted** **OR**	**95% CI**	**p-value**	**n(%) poor treatment outcomes**	**Unadjusted** **OR**	**95% CI**	**p-value**
		
***Distal level***								
		
Sex								
Female	29/152(19.1%)	1	-	0.10	0/6 (0%)	1	-	.
Male	9/75 (12.0%)	0.58	0.26-1.29		47/111(42.3%)	.	.	
		
Age (years)								
<35	18/96(18.8%)	1	-	0.63*	10/23(43.5%)	1	-	0.28*
34-45	11/81(13.6%)	0.68	0.30-1.54		7/26(26.9%)	0.48	0.15-1.58	
>45	9/50(18.0%)	0.95	0.39-2.30		30/68(44.1%)	1.02	0.40-2.66	
		
Education (years)								
0-6	6/36(16.7%)	1	-	0.85*	31/55(56.4%)	1	-	<0.001*
7-10	14/75(16.7%)	1.15	0.40-3.28		11/45(24.4%)	0.25	0.11-0.59	
>10	18/116(15.5%)	0.92	0.33-2.52		5/17(29.4%)	0.32	0.10-1.04	
		
Alcohol consumption/week (units)								
No alcohol	27/183(14.8%)	1	-	0.02^† ^	29/69(42.0%)	1	-	0.87
1-20 units	6/32(18.8%)	1.33	0.50-3.54		8/22(36.4%)	0.79	0.29-2.12	
>21 units	5/11(45.5%)	4.81	1.37-16.89		10/26(38.5%)	0.86	0.34-2.17	
		
Time since first HIV test in community programme (months)^a^								
<6	10/74(13.4%)	1	-	0.27*	n.a.	n.a.	n.a.	n.a.
6-24	10/72(13.9%)	1.03	0.40-2.65					
>24	18/81(22.2%)	1.82	0.78-4.27					
		
Time since first HIV test in workplace programme (months)^a^								
<0.5	n.a.	n.a.	n.a.	n.a.	10/19(52.6%)	1	-	0.02
0.5-3					13/47(27.7%)	0.34	0.11-1.04	
>3					22/46(47.8%)	0.83	0.28-2.41	
		
								
		
Know anyone who died of HIV								
Yes	28/168(16.7%)	1	-	0.79	25/80(31.3%)	1	-	<0.001
No/not sure	8/53(15.1%)	0.89	0.38-2.09		22/36(61.1%)	3.46	1.52-7.85	
		
Know someone on ART								
No	26/144(19.6%)	1	-	0.18	25/57(43.9%)	1	-	0.33
Yes	12/94(12.8%)	0.60	0.29-1.26		7/22(31.8%)	0.60	0.21-1.69	
		
Informed that being tested for HIV								
Yes	31/201(15.4%)	1	-	0.17	44/114(38.6%)	1	-	.
No/not sure	7/26(26.9%)	2.02	0.78-5.21		1/1(100.0%)	.	.	
		
Satisfied with programme services								
Agree or strongly agree	34/173(19.7%)	1	-	0.06^‡^	16/57(28.1%)	1	-	0.01^‡^
Disagree or strongly disagree	0/16(0%)	.	.		6/8(75.0%)	7.78	1.40-42.14	
Don't know	4/38(10.5%)	0.48	0.16-1.45		25/52(48.1%)	2.37	1.10-5.25	
		
***Intermediate level***								
		
A healthy looking person can have HIV								
Yes	32/206(15.5%)	1	-	0.15	22/80(27.5%)	1	-	0.001
Not/not sure	6/21(28.6%)	2.18	0.79-6.03		22/34(64.7%)	4.83	2.05-11.49	
		
Belief in HIV existence								
Yes	36/224(16.1%)	1	-	0.07^‡^	36/102(35.3%)	1	-	0.02
No/not sure	2/3(66.8%)	10.44	0.92-118.25		11/14(78.6%)	6.72	1.76-25.66	
		
Belief in own HIV status								
Yes	35/221(15.7%)	1	-	0.06^‡^	37/102(36.3%)	1	-	0.03
No/not sure	3/6(50.0%)	5.31	1.03-27.41		10/15(66.7%)	3.51	1.12-11.06	
		
A traditional healer can treat HIV								
Disagree/strongly disagree	24/149(16.1%)	1	-	0.76^‡^	17/63(27.0%)	1	-	0.01*
Agree/strongly agree	2/9(22.2%)	1.49	0.29-7.60		10/18(55.6%)	3.38	1.14-10.00	
Not sure/don't know	12/169(17.4%)	left1.10	0.51-2.35		20/36(55.6%)	3.38	1.43-8.00	
		
Consulted traditional healer in past year								
No	25/170(14.7%)	1	-	0.15	22/60(36.7%)	1	-	0.43
Yes	13/56(23.2%)	1.75	0.83-3.72		25/57(43.9%)	1.35	0.64-2.83	
		
***Proximate level***								
		
Effect of ART on health								
Feel better	22/134(16.4%)	1	-	0.85	26/87(29.9%)	1	-	<0.001
Not sure/don't know	16/92(17.4%)	1.07	0.53-2.17		21/30(70.0%)	5.47	2.21-13.54	
		
ART can cure HIV								
No	23/153(15.0%)	1	-	0.33	21/65(32.3%)	1	-	0.06
Yes/not sure	15/74(20.3%)	1.44	0.70-2.95		25/50(50.0%)	2.10	0.98-4.48	
		
How difficult or easy will it be to adhere?								
Easy/very easy	36/197(18.3%)	1	-	0.08^‡^	24/76(31.6%)	1	-	0.01
Difficult/very difficult/not sure	2/30(6.7%)	0.32	0.07-1.40		23/41(56.1%)	2.80	1.30-6.10	
		
Disclosed HIV status								
Yes	36/222(16.2%)	1	-	0.20^‡^	18/56(32.1%)	1	-	0.08
No	2/5(40.0%)	3.44	0.56-21.35		29/60(49.3%)	1.97	0.93-4.20	
		
Disclosed the initiation of ART								
Yes	29/196(14.8%)	1	-	0.09	9/37(24.3%)	1	-	0.02
No	8/29(27.5%)	2.19	0.89-5.42		38/80(47.5%)	2.81	1.17-6.71	
		
Social support for taking ART								
Yes	37/222(16.7%)	1	-	1.00^‡^	13/39(33.3%)	1	-	0.16
No/not sure	1/5(20.0%)	1.25	0.14-11.50		20/41(48.8%)	1.90	0.77-4.71	
		
Prior TB treatment								
No	25/117(21.4%)	1	-	0.06	24/59(40.7%)	1	-	0.91
Yes	13/109(11.9%)	0.50	0.24-1.03		8/19(42.1%)	1.06	0.37-3.03	
		
Adherence to prior chronic treatment								
Yes	16/135(11.9%)	1	-	0.06*	21/51(41.2%)	1	-	0.85^‡^
No	7/29(24.1%)	2.40	0.87-6.41		26/66(39.4%)	0.93	0.44-1.96	.
Not prescribed prior treatment	15/63(23.8%)	2.30	1.07-5.07		0	.	.	
		
Sharing medicines with family and friends								
Never/rarely	37/212(17.5%)	1	-	0.4^‡^	18/62(29.0%)	1	-	0.01
Sometimes/often/always	1/14(7.1%)	0.36	0.04-2.87		25/47(53.2%)	2.78	1.26-6.14	
		
***Biological variables***								
		
CD4 count at baseline (cells/mm3)								
<100	18/111(16.2%)	1	-	0.78*	8/24(33.3%)	1	-	0.10*
100-200	18/99(18.2%)	1.15	0.56-2.35		24/46(52.2%)	2.18	0.78-6.09	
>200	2/17(11.8%)	0.69	0.14-3.28		15/47(31.9%)	0.94	0.33-2.67	
		
Viral load at baseline (copies/ml)								
<100,000	21/105(20.0%)	1	-	0.22	33/79(41.8%)	1	-	0.61
≥ 100,000	17/122(13.9%)	0.65	0.32-1.31		14/38(36.8%)	0.81	0.37-1.80	

OR: Odds ratio; CI: Confidence interval

*P value from likelihood ratio test

^† ^P value for trend

^‡^P value from Fisher's exact test

^a ^Due to the large variation in the distribution of time since first HIV test between the two programmes this variable was categorised differently for each programme.

The results of the logistic regression analysis are shown in additional file 1 (table S1). In the community programme, in the final model, in which proximate factors were adjusted for the confounding role of intermediate and distal variables, factors associated with better treatment outcomes were: being male (adjusted [a] odds ratio [OR] 0.25, 95% CI 0.09-0.74) and knowing someone on ART (aOR 0.44, 95% CI 0.19-1.01). Drinking more than 20 units of alcohol per week, having no prior experience of taking chronic medications, and consulting a traditional healer in the past year were associated with worse outcomes (aOR 15.36, 95%CI 3.22-73.27; aOR 2.30, 95%CI 1.0-5.3; aOR 2.27, 95%CI 1.00-5.19 respectively) (see Table S1).

In the workplace programme, factors associated with poor treatment outcomes were: being uncertain regarding a traditional healer's ability to treat HIV (aOR 4.40, 95%CI 1.41-13.75); being uncertain about the health effects of ART (aOR 7.53, 95%CI 2.02-27.98); and more frequently sharing medicines with family and friends (aOR 3.46, 95%CI 1.26-9.50). Having 2 weeks to 3 months pre-ART waiting time compared to having less than 2 weeks pre-ART waiting time was associated with better treatment outcomes (aOR 0.13, 95%CI 0.03-0.56) (see Table S1).

### Sensitivity analyses

In a sensitivity analysis restricted to individuals with a viral load result at the six-month visit, and in which the outcome was defined as a viral load of >400 copies/ml, the associations between exposures and the outcome were generally similar in trend and magnitude to those in the primary analysis for both programmes. However, in the workplace, a pre-ART waiting time of less than two weeks was more strongly associated with lack of virological suppression in the sensitivity analysis than with the composite outcome of non-suppression and discontinuation of treatment in the primary analysis (aOR 0.04, 95%CI 0.01-0.28; aOR 0.13, 95%CI 0.03-0.56 respectively).

A second sensitivity analysis was conducted in which factors strongly predictive of poor treatment outcomes were reclassified in the hierarchical framework to determine if and how this would affect the final models. In the community programme, traditional medicine use was reclassified as a distal factor while time since known HIV status and knowing someone ART were reclassified as intermediate factors. In the workplace programme, beliefs regarding HIV and beliefs regarding traditional medicines were reclassified as distal factors, while CD4 count at baseline, knowing someone with HIV and time of known HIV status were reclassified as intermediate factors. There were no important differences between the final model obtained from the primary analysis and that obtained from the sensitivity analysis in either programme (data not shown).

## Discussion

After six months on ART, poor treatment outcomes were more common among participants in the longer-established and better-resourced workplace programme than in the community programme. The predictors of poor outcomes were largely unique to each programme, with non-biomedical beliefs concerning HIV and ART playing a larger role in the workplace programme than in the community programme.

The workplace programme may have features in common with others that offer increased access to the catchment ART population through robust VCT, PITC, and the minimization of structural barriers known to inhibit access to treatment. A paradoxical consequence of the success in promoting HIV testing and facilitating access to care in the workplace programme, such that an estimated 95% of those eligible have started ART (C Innes, personal communication), may partially explain the poorer treatment outcomes observed. This approach likely extended ART to a "second generation" [23,24] of patients who, without the active promotion of PITC and referral for treatment, may have been less motivated to self-refer to these services. Uncertainty regarding the health benefits of ART, previously reported to inhibit adherence in other lower-income settings [25-30], was prevalent and highly predictive of poor treatment outcomes in the workplace. In this second generation of potentially less well-motivated patients we can hypothesise that beliefs regarding ART efficacy may play a larger role in determining adherence than structural barriers, which are critical among those who seek out treatment more spontaneously in the community programme. Among individuals entering care through PITC and referral, there may be a need for more robust adherence support focused not only on ameliorating structural barriers to succeeding on treatment, but also on addressing alternative beliefs regarding HIV and its treatment.

Uncertainty regarding ART benefits in the workplace programme may also have been in part due to a "healthy worker" effect such that workplace participants, who were more likely to feel physically well, were also more sceptical regarding the necessity for ART [31]. It also may have reflected the surprisingly prevalent scepticism regarding the existence of HIV altogether among workplace participants [19], which could in part relate to lower levels of education or the rural origin of many employees, where alternative beliefs about disease causation may be more robust than in urban areas.

Positive attitudes towards the role of traditional healers, associated with poor adherence in other studies from lower-income countries [13,32,33], were predictive of poor treatment outcomes in both programmes. Seeking advice or treatment from traditional healers is commonplace throughout Africa [34] and individuals seeking health care may oscillate between representatives of western biomedicine and traditional healers, without much tension or sense of contradiction [35]. However, in the case of HIV treatment, both western and traditional practitioners may be likely to advise their patients to not mix ART with traditional medicines, thereby leading some patients to discontinue ART either temporarily or permanently. Given the ubiquitous role of traditional healers in providing first-line care for HIV-infected individuals, counselling paradigms must evolve to include training for western medicine practitioners on counselling patients regarding adherence to ART even if they elect to seek alternative methods of healing while on ART.

Past treatment-taking experience was predictive of poor treatment outcomes in both programmes. In the community programme, prior adherence to chronic treatment predicted good treatment outcomes. Reported adherence to prior chronic treatment may indicate an increased level of self-efficacy regarding one's own ability to adhere to long-term treatment, a factor previously linked to better adherence [25,27,36,37]. In the workplace programme, a history of treatment sharing with family and friends was predictive of poor treatment outcomes. Treatment sharing may have been more prevalent in this setting where family members had no immediate access to the workplace ART programme. Efforts in the workplace programme to increase ART access to partners of ART patients may play an important role in improving adherence.

In the workplace programme, starting ART less than two weeks after being diagnosed with HIV was highly predictive of poor treatment outcomes. Individuals who started ART less than two weeks after HIV diagnosis represented those who were referred to the HIV programme for pre-ART counselling on the same day they were tested for HIV or shortly thereafter, and were then started on ART on the same day they were confirmed to be eligible for treatment or shortly thereafter. Rapid ART initiation partly reflects a strong commitment to improving access to ART among those eligible for treatment in the workplace programme. However, this study highlights the challenge of providing adequate pre-ART counselling support when individuals need to start ART urgently (less than 2 weeks after HIV diagnosis). Thus, in this and similar settings there may be a need for careful balance between efforts to minimise delays in ART initiation, once medically required, and allowing sufficient time for the provision of adequate counselling and adherence preparation.

## Limitations

In the absence of a gold standard for measuring adherence to ART, viral load was used as a proxy measure of adherence. It remains possible for a limited number of individuals to have been adherent to highly efficacious ART regimens but to not have achieved virological suppression at six months. However, the potential for this to affect the estimate of adherence in this study was limited by the fact that the cohort consisted of treatment-naïve participants starting treatment at a time when ART availability was restricted and among whom transmitted resistance is unlikely [38-44]. In this population, viral load was arguably the best measure of adherence [4,5,45-48]. Participants were referred to the study by clinic staff and we do not have information on the number of people starting ART in the clinic who were eligible for the study but refused referral. Therefore, we cannot be certain that the prevalence of specific characteristics is representative of all patients in the clinic who were eligible for the study. Nonetheless, the study analysis compared data within the cohort and thus was valid in ascertaining which characteristics were associated with the outcomes observed. This is especially true considering that the refusal rates once patients were offered study participation were very low (0% in the community and 2% in the workplace programme). The programme-specific analysis enabled the identification and the exploratory comparison of several important and strongly associated predictors of poor treatment outcomes. While these findings allowed for a discussion of the potential differences between predictors of adherence in the two programmes, these differences were not anticipated and thus the study was not designed nor powered to compare them formally.

## Conclusions

Poor treatment outcomes were more common in the well-resourced workplace programme and largely predicted by different factors in comparison with the community programme. This difference reflects the potential for the community programme to have a primarily self-selected and hence a more motivated and a more adherent patient population, among whom structural factors are the main barriers to adherence and subsequent success on treatment. In contrast, the workplace programme may have a less selected, and hence less motivated and less adherent patient population, among whom non-biomedical beliefs of illness and treatment play a larger role in predicting outcomes. In the workplace and similar programmes in lower income-countries, where PITC extends ART to individuals potentially less motivated to seek care, patients may need additional adherence support, especially addressing uncertainty about the health benefits of ART and how to maintain adherence even if patients elect to seek complementary methods of treatment. Meanwhile, in the community programme and in others that are similarly less focused on PITC, efforts to improve adherence and retention should continue to prioritize addressing structural barriers to treatment and increasing access to ART to those currently unable to receive treatment.

## Competing interests

The authors declare that they have no competing interests.

## Authors' contributions

MD: protocol development, data collection, analysis and manuscript development; SC: support with protocol development, supervision of data collection and review of manuscript; AK: support with data collection and review of manuscript; KF: support with protocol development, data analysis and review of manuscript; RH: support with protocol development, data collection and review of manuscript; GJC: support with protocol development, data collection and review of manuscript; AG: support with protocol development, data collection, analysis and manuscript development. All authors have read and approved the final manuscript.

## Appendix 1: Description of hierarchical framework used for organising the multivariable regression analysis

In the hierarchical conceptual framework used to conduct multivariable analysis, factors of interest were organized into three levels according to how directly they were thought to influence adherence behaviour [22]. Distal level exposures of interest were factors thought to affect adherence behaviour indirectly or through other mediating factors. These primarily included personal characteristics and experiences of health-care services. Intermediate level exposures of interest were factors thought to mainly affect adherence through more proximate determinants. These intermediate level factors primarily included factors related to beliefs regarding HIV and traditional medicines. Finally proximate exposures of interest were ones thought to more directly influence adherence behaviour. This included factors related to beliefs in ART, disclosure and adherence to prior chronic treatment. This subdivision facilitated the organization of variables according to their theoretical hierarchical relationship to each other and to the treatment outcome. Organising variables using these theoretical hierarchical relationships was deemed important for two reasons: firstly, to avoid the misclassification of proximate factors as confounders of the distal determinants and the reduction or nullification of the true effect of the more distal determinants [22], and secondly, the hierarchical classifications enabled the grouping of similar variables into the same hierarchical level in order to facilitate the assessment of co-linearity among variables.

### Steps in the multivariable analysis

Variables found to be associated with the outcome in the univariable analysis (p < 0.2) and any potential confounders were further grouped into three hierarchical levels. Figure 1 illustrates the hierarchical multivariable analysis approach used to identify determinants of poor adherence at each hierarchical level. Firstly distal level exposures of interest were analysed, starting with a full model that included all distal level exposures found to be associated with the outcome in the univariable analysis (p < 0.2) and any potential confounders. Explanatory variables were then sequentially dropped from the model, starting with variables with the weakest association with the outcome based on the likelihood ratio test. Only variables found to be associated with the outcome at a p-value smaller than 0.1 and *a priori *confounders were retained. Secondly, the full intermediate level model included distal level determinants identified in the previous model, intermediate level exposures found to be associated with the outcome in the univariable analysis (p < 0.2) and any *a priori *confounders. Finally at the proximate level, exposures of interest were analysed starting with a full model that included distal level determinants, intermediate level determinates, all proximate level exposures found to be associated with the outcome in the univariable analysis (p < 0.2) and any *a priori *confounders.

At each level, variables thought to be highly correlated within each group were cross-tabulated in order to investigate co-linearity. Two tests for co-linearity, tolerance and variance inflation factor (VIF), were calculated to test the strength of the interrelationships among the variables. Tolerance is an indicator of how much co-linearity a regression analysis can tolerate, while VIF is an indicator of how much of the inflation of the standard error could be caused by co-linearity [49]. If co-linear variables were identified, one variable, that with the strongest association with outcome, was chosen to represent the co-linear variables in the group.

Explanatory variables were then sequentially dropped within each group, starting with variables with the weakest association with the outcome based on the likelihood ratio test. Only variables found to be associated with the outcome at a p-value < 0.1 were retained. This threshold was chosen (as opposed to the more conventional 0.05) to minimise the chances of erroneously excluding potentially important risk factors [50,51]. However, the interpretation of the models considered not only the magnitude of the association (size of the odds ratios) but also the precision of the point estimates (width of the confidence intervals) [50]. The model presented is the final model in which the effects of proximate variables are adjusted for the confounding role of distal and intermediate variables.

### Sensitivity analysis

Since there may be more than one logical way in which variables could be grouped, a sensitivity analysis was conducted to test the potential effect on regression analysis of employing an alternative hierarchical framework. In this alternative conceptual framework, key factors associated with poor adherence in the primary regression analysis were reclassified at different levels in the hierarchy to test if and how this would change the models obtained from the primary regression analysis. For example, beliefs in HIV and traditional medicines were reclassified as distal factors, and factors related to education and to income were reclassified as intermediate factors. Factors related to beliefs regarding ART and experience with chronic medications remained classified as proximate factors.

## Pre-publication history

The pre-publication history for this paper can be accessed here:

http://www.biomedcentral.com/1471-2458/10/430/prepub

## Supplementary Material

Additional file 1 **Table S1: Baseline predictors of poor treatment outcomes (viral load >400 copies/ml and complete discontinuation of follow-up and treatment six months post treatment initiation) on antiretroviral therapy: univariable and multivariable adjusted models**. Table S1 shows the multivariable adjusted analysis of baseline variables associated with poor treatment outcomes (VL >400 copies/ml or complete discontinuation of follow-up and treatment) after six months of treatment. The multivariable analysis was conducted using a hierarchical framework that grouped exposures into three main levels: distal, intermediate and proximate, depending on how directly they were thought to influence the outcome. The framework served to better organise the analysis in order to avoid the misclassification of proximate factors as confounders of the distal ones, and to enable the grouping of similar variables into the same hierarchical level in order to assess co-linearity. Click here for file
