# Supplementary material for: Contrasting predictors of poor antiretroviral therapy outcomes in two South African HIV programmes: a cohort study
Source: BMC Public Health. 2010 Jul 22;10:430. doi: 10.1186/1471-2458-10-430 (PMC2920888; doi:10.1186/1471-2458-10-430)
Supplement: Additional file 1 — Table S1: Baseline predictors of poor treatment outcomes (viral load >400 copies/ml and complete discontinuation of follow-up and treatment six months post treatment initiation) on antiretroviral therapy: univariable and multivariable adjusted models. Table S1 shows the multivariable adjusted analysis of baseline variables associated with poor treatment outcomes (VL >400 copies/ml or complete discontinuation of follow-up and treatment) after six months of treatment. The multivariable analysis was conducted using a hierarchical framework that grouped exposures into three main levels: distal, intermediate and proximate, depending on how directly they were thought to influence the outcome. The framework served to better organise the analysis in order to avoid the misclassification of proximate factors as confounders of the distal ones, and to enable the grouping of similar variables into the same hierarchical level in order to assess co-linearity. [file 1471-2458-10-430-S1.DOC]

**Table S1: Baseline predictors of poor treatment outcomes (viral load >400 copies/ml or complete discontinuation of follow-up and treatment six months post treatment initiation) on antiretroviral therapy: univariable and multivariable adjusted models**

| **Variable** |  | **Unadjusted OR**  **(95% CI)** |  | **Distal model^a^**  **Adjusted OR**  **(95% CI)** |  | **Intermediate model^b^**  **Adjusted OR**  **(95% CI)** |  | **Proximate model^c^**  **Adjusted OR**  **(95% CI)** |  | **Sensitivity analysis of proximate model^d^ Adjusted OR**  **(95% CI)** |
| --- | --- | --- | --- | --- | --- | --- | --- | --- | --- | --- |
| **Community Programme** | | | | | | | | | | |
| ***Distal*** |  |  |  |  |  |  |  |  |  |  |
| Gender  Female  Male |  | 1  0.58 (0.26-1.29) |  | 1  0.25 (0.09-0.71) |  | 1  0.22 (0.08-0.64) |  | 1  0.25 (0.09-0.74) |  | 1  0.34 (0.10-1.15) |
| Know someone on ART  No  Yes |  | 1  0.60 (0.29-1.26) |  | 1  0.42 (0.19-0.94) |  | 1  0.40 (0.18-0.90) |  | 1  0.44 (0.19-1.01) |  | 1  0.64 (0.25-1.65) |
| Alcohol consumption  Non-drinker  1-20 units/week  >20 units/week |  | 1  1.33 (0.50-3.54)  4.81 (1.37-16.89) |  | 1  2.21 (0.74-6.57)  14.69 (3.16-68.26) |  | 1  2.06 (0.69-6.18)  14.50 (3.12-67.37) |  | 1  2.08 (0.68-6.34)  15.36 (3.22-73.27) |  | 1  2.74 (0.81-9.22)  7.53 (1.04-54.55) |
| ***Intermediate*** |  |  |  |  |  |  |  |  |  |  |
| Consulted a traditional healer in past year  No  Yes |  | 1  1.75 (0.83-3.72) |  |  |  | 1  2.17 (0.96-4.90) |  | 1  2.27 (1.00-5.19) |  | 1  1.38 (0.49-3.91) |
| ***Proximate*** |  |  |  |  |  |  |  |  |  |  |
| Adherence to prior chronic treatment (tx)  Yes  No  Not prescribed prior treatment |  | 1  2.4 (0.87-6.41)  2.3 (1.07-5.07) |  |  |  |  |  | 1  2.13 (0.74-6.12)  2.30 (1.00-5.30) |  | 1  1.77 (0.50-6.33)  1.92 (0.72-5.13) |
| **Workplace Programme** | | | | | | | | | | |
| ***Distal*** |  |  |  |  |  |  |  |  |  |  |
| Education in years  0-6  7-10  >10 |  | 1  0.25 (0.11-0.59)  0.32 (0.10-1.04) |  | 1  0.23 (0.12-0.90)  0.29 (0.07-1.20) |  | 1  0.31 (0.11-0.85)  0.38 (0.10-1.39) |  | - |  | - |
| Time since first HIV test in months  <0.5  0.5-3  >3 |  | 1  0.34 (0.11-1.04)  0.83 (0.28-2.41) |  | 1  0.15 (0.04-0.59)  0.63 (0.20-2.06) |  | 1  0.16 (0.04-0.60)  0.48 (0.14-1.71) |  | 1  0.13 (0.03-0.56)  0.42 (0.11-1.64) |  | 1  0.04(0.01-0.28)  0.09(0.02-0.58) |
| Know anyone who died of HIV  Yes  No/not sure |  | 1  3.46 (1.52-7.85) |  | 1  2.41 (0.90-6.43) |  | -^*^ |  | -^*^ |  | -^*^ |
| Satisfied with programme services  Agree or strongly agree  Disagree or strongly disagree  Don’t know |  | 1  7.78 (1.40-42.14)  2.37 (1.10-5.25) |  | 1  21.2 (1.69-266.48)  2.17 (0.86-5.48) |  | -^*^ |  | -^*^ |  | -^*^ |
| ***Intermediate*** |  |  |  |  |  |  |  |  |  |  |
| Belief in existence of HIV  Yes  No/not sure |  | 1  6.72 (1.76-25.66) |  |  |  | 1  5.80 (1.31-26.1) |  | 1  2.82 (0.55-14.53) |  | 1  2.13 (0.21-21.35) |
| Traditional healer can help me treat HIV  Disagree/strongly disagree  Agree/strongly agree  Not sure/don’t know |  | 1  3.38 (1.14-10.00)  3.38 (1.43-8.00) |  |  |  | 1  3.16 (0.85-11.77)  3.08 (1.08-8.77) |  | 1  1.81 (0.38-8.56)  4.40 (1.41-13.75) |  | 1  1.29 (0.12-13.71)  6.23 (1.26-30.91) |
| ***Proximate*** |  |  |  |  |  |  |  |  |  |  |
| Effect of ART on health  Feel better  Not sure/don’t know |  | 1  5.47 (2.21-13.54) |  |  |  |  |  | 1  7.53 (2.02-27.98) |  | 1  14.75(1.95-111.40) |
| History sharing medicines with family/friends  Never/rarely  Sometimes/often/always |  | 1  2.78 (1.26-6.14) |  |  |  |  |  | 1  3.46 (1.26-9.50) |  | 1  2.03 (0.53-7.74) |

^a^ Overall effect of each distal variable adjusted for each other but not adjusted for mediating variables

^b^ Effect of intermediate variables adjusted for confounding role of distal variables

^c^ Final model with proximate variables adjusted for confounding role of distal and intermediate variables

^d^ Poor treatment outcome restricted to having a viral load >400 copies/ml

*Excluded variable because it did not improve the model’s fit
